# Complete genome sequencing and probiotic characterization of promising lactic acid bacterial strains isolated from dairy products in Egyptian markets

**DOI:** 10.1186/s12866-025-03757-3

**Published:** 2025-02-06

**Authors:** Mostafa F. El-Hosseny, Mohamed G Seadawy, Mohammed O Abdel-Monem, Mervat G Hassan

**Affiliations:** 1 Biodefense Center for Infectious and Emerging Diseases, Ministry of Defense, Cairo, Egypt; 2 Botany and Microbiology Department, Faculty of Science, Banha University, Banha, Egypt

**Keywords:** Probiotics, Dairy products, Complete genome sequencing, Human health, CRISPR

## Abstract

**Background:**

Probiotics refer to live bacteria that, when administered in a sufficiently, exert a beneficial influence on human health. Due to the probiotics' beneficial health advantages, dietary supplements are expanding rapidly as a self-care interest worldwide. It may be beneficial to administer probiotic strains resistant to antibiotics concurrently with an antibiotic treatment. Our study investigates nineteen dairy products collected from Egyptian markets, isolated, identified and underwent a characterization for probiotic features under demanding circumstances as NaCl, acid and bile salt environments. The antibiotic sensitivity test was performed later to the antimicrobial assessment against widespread both negative and positive gram-stained bacteria infecting humans, along with the antiviral evaluation against (*SARS-CoV-2*), the virus that has disturbed the world recently.

**Results:**

Out of nineteen investigated isolates, five potential probiotic isolates were examined for probiotic characteristics. Our tested samples were of dairy origin (yogurt, cottage-cheese and sour milk) in Egypt, were identified as *Lactobacillus delbrueckii* subsp. *bulgaricus, Streptococcus thermophilus* and *Pediococcus acidilactici*. These promising isolates had withstood stressful factors, such as NaCl, acid, bile salts, and the antimicrobial advance. The genomes were characterized for the physiology, safety, and efficacy of these isolates for probiotic qualities plus the presence of mobile genetic components and prophages that influence the genome's flexibility. They lack virulence factors and pathogenicity, rather than the lack of antibiotic resistance genes.

**Conclusion:**

Three promising isolates underwent complete genome sequencing with high-throughput second generation technology followed by comprehensive bioinformatic analysis. The results showed that our isolates possess traits enabling resilience to antimicrobial effects and stress factors that might cause problems in the human gut. Several trustworthy genomic analysis methods were used to confirm and provide detailed illustrations of all traits. Genomic analyses confirmed the presence of stable genomes due to including mobile genetic components such as phages and CRISPR clusters, which validate their quality and safe usage for human health.

## Introduction

Living bacteria classified as probiotics, can benefit human health when taken in the appropriate amounts [1]. The bacterial strain can be classified as a probiotic when satisfies the criteria for safety, functionality, and technological applicability. The safety criteria include human or animal origin; isolation from the alimentary tract of healthy individuals; confirmation of a safe use record; and absence of infectious disease potential [2]. The strain must meet the functional requirements of being resistant to the bacteriocins, acids, and bile salts produced by gut microbiota. Additionally, it must survive, metabolize, grow in the gut environment, and resist pathogenic colonization. Probiotics are technologically beneficial when their genomes are stable, resistant to bacteriophages, and end products are viable and durable throughout processing and storage [2]. Probiotics are recommended both before and after antimicrobial treatment to help restore the body's natural microbiota, support digestion, enhance nutrient and vitamin absorption, and alleviate food allergies [3]. Furthermore, probiotics are utilized to support the treatment of obesity, improve of lipid profiles and carbohydrate metabolism, and enhance host immunity [4]. Lactic acid bacteria (LAB) are a particularly prevalent, widely dispersed, and frequent resident of the human gut epithelium among the probiotic microbiota under investigation. Dairy products are rich source of LAB like processed yogurt and cottage-cheese, which is a fresh, un-ripened cheese made from milk, has a mild, tangy flavor and a soft creamy texture and is a good source of protein and calcium. LAB are a diverse group of Gram-positive bacteria that play a crucial role in various aspects of human life, particularly in food production and human health. Along with the human health benefits of LAB like gut health and immunity improvements, they have importance in food production as fermentation, preservation, flavor and texture [5].Many LAB species are widely employed in important traditional and industrial fermented dairy products, particularly *Lactobacillus delbrueckii* subsp. *bulgaricus, Streptococcus thermophilus* and *Pediococcus acidilactici* [6]. *L. delbrueckii* is a gram-positive, non-sporulating, cocco-bacilli or rod-shaped bacteria with a typical GC genome content of less than 50% [7]. *Streptococcus thermophilus* is gram-positive, non-motile and non-endospore-forming small, long chains of cocci that withstand elevated temperatures with a GC content less than 50% [8]. *Pediococcus acidilactici* is gram-positive paired or tetrad cocci, and can grow in a wide range of pH, temperature, and osmotic pressure [9]. These LAB were utilized as starter cultures in the production of various fermented dairy products, including cheese and yoghurt [10], and have been generally recognized as safe (GRAS) by the Food and Drug Administration (FDA) [11]. Moreover, because it is dairy-derived, making its recipes for consumption would be easier.

Our study aims to investigate these local dairy isolates for their probiotic category through biochemical, stress endurance and molecular analyses which is further crowned by complete sequencing. Whole genome sequencing (WGS) of these isolates will serve as the foundation for *in vitro* safety evaluations, which may be verified by *in vivo* studies for pharmaceuticals intended for human use. WGS sheds light on the species' biology and helps identify genes unique to a particular strain, as well as its transcripts, probiotic attributes, and antimicrobial properties.

## Methodology

### Sampling and isolation

Nineteen milk-product samples (milk, sour milk, yogurt, and cottage cheese) were gathered from different Egyptian markets. We obtained 50 gm from each sample as 5 samples yogurt, 5 samples sour milk, 5 samples cottage-cheese and 4 samples raw milk. After packing and marking, the collected samples were immediately transferred in polyethylene bags on ice to the Main Chemical War laboratory near Nasr City in Cairo, Egypt [12]. Samples were homogenized by stomacher mixer in 10 mL buffered peptone water, incubated for 18 h at 37°C then, 1mL of the non-selective buffered peptone suspension was added to the de Man-Rogosa-Sharpe MRS broth (Merck, Germany) as enrichment media and incubated for 24 h at 37°C. MRS agar (Merck, Germany) plates were inoculated by 0.1ml grown samples and incubated at 37°C for 48h anaerobically (anaerobic jar and gas pack) [13].

### Phenotypic characterization

To perform phenotypic characterization (morphological and biochemical), sub-culturing of single clear positive colonies was done on MRS agar plates three successive times for purification. Colonial morphology (colony shape, color, margin and opacity); gram staining and microscopic examination were inspected for each isolated culture [14]. Biochemical confirmation were done using the VITEK® 2C automated platform (bioMérieux, France) and the recommended ANC cards per the manufacturer's guidance [15].

### Characterization for probiotic criteria

Confirmed gram-positive isolates were estimated for tolerance to NaCl, acids, bile salt, antibiotics and capability to suppress selected pathogens. According to the recommended procedure M7-A7-CLSI of Chemical Laboratory Standard Institute [16], each isolate's inoculum was made in tubes with 5 ml of broth media and incubated for 24h at 37°C. The inoculum's turbidity was sustained at 0.5 McFarland with 10^7^−10^8^ CFU/ml.

### Tolerance to sodium chloride (NaCl)

NaCl tolerance was determined according to Uymaz Tezel et al. [17] with some modifications. Modified MRS broth (Merck, Germany) with different NaCl concentrations (0%, 3%, 4%, 5%, 6%, and 7%) (w*/*v) was applied. To the freshly prepared overnight culture, 1% (v/v) isolate was added into modified MRS and incubated anaerobically at 37ºC for (2–5) h intervals. The pure MRS broth acted as a control group, and the trial was performed in tripartite. At 600 nm, the absorbance was determined following two and five hours.

### Acid and bile tolerance

As illustrated by Shaikh & Shah [18], isolates were investigated for viability an acidic medium. Overnight (1.3x 10^9^ CFU/ml) cultures in MRS broth were spun down at 5000 rpm for 10 min. After re-suspension of pellets in the PBS buffer which was modified at pH (3, 4, 5, 6) and pH 7 (control) and incubation at 37°C for 2h and 5h, 1% from each trial was added to new MRS broth (pH 7) and incubated for 24h at 37°C in tripartite pattern. The growth was measured at OD_600_. Isolates with more than 50% resistance at pH 4, were designated acid-tolerant ones.

Each isolate was evaluated for tolerance to bile salt according the Vinderola and Reinheimer's approach [19]. Simply 200 µl from each isolated inoculum suspension (10^7^−10^8^ CFU/ml) were inoculated into 1000 µl of MRS broth with different doses of bile salt (0.1, 0.2, 0.3, 0.4 and 0.5% w/v) plus control MRS broth without bile salt and incubated for 2h and 5h at 37 C. Following incubation, the OD (optical density) was measured at 600 nm for comparison with the control. Isolates with more than 50% resistance at 0.3% bile concentration were designated bile-tolerant culture. The percent of tolerance was estimated in both circumstances (acid pH / bile values) as:

$${\%}\,\text{R}\text{e}\text{s}\text{i}\text{s}\text{t}\text{a}\text{n}\text{c}\text{e}=\frac{Increment\:of\:OD\:in\:MRS\:broth\:with\:bile\:salt/pH3,\:\text{4,5},\:6\:}{Increment\:of\:OD\:in\:MRS\:broth\:with\:bile\:salt/pH\:7}\times\:100$$

### Antibiotics susceptibility

Using the disc diffusion technique, antibiotic sensitivity for each isolate was evaluated on Mueller-Hinton agar (MHA) [20]. The turbidity of the nocturnal tested inoculums (10^7^−10^8^ CFU/ml) was adjusted to 0.5 McFarland. MHA with methylene blue (0.5 g/ml) supplemented by 5% defibrinated horse blood and was inoculated by each isolate. BD BBL™ Sensi-Disc™ antibiotic susceptibility discs used in the test were (Ampicillin, Azithromycin, Ceftriaxone, Chloramphenicol, Ciprofloxacin, Clarithromycin, Erythromycin, Gentamicin, Nalidixic acid, Neomycin, Penicillin, Streptomycin, Tetracycline, Trimethoprim and Sulfamethizole). All discs were located on these streaked cultures by sterilized forceps, tested dishes were refrigerated for 2h and then incubated for 20h at 35°C. The zone of inhibition (ZOI) was measured according to the Laboratory and Clinical Standard Institute's guidelines [21].

### Antibacterial activity

Antibacterial activity of all isolates against gram-negative and gram-positive pathogens (*S. aureus, S. typhimurium, K. pneumonia, E. coli, E. fecalis, L. monocytogens)* was assessed on MHA plates using the standard well diffusion procedure [22]. These tested pathogenic bacteria were obtained from the biobank of Main Chemical Warfare Laboratory. Each MHA plate was spread by 100 µl of pathogen inoculum (10^7^−10^8^ CFU/ml). Wells in each infected culture were inoculated by 30 µl cell-free solution obtained by centrifugation of 24h grown inoculum in MRS broth at 4000 rpm for 10 min. The negative control used was MRS broth, while the positive control was ciprofloxacin (30 ug/ml). To ensure that the suppression is not caused by lactic acid instead of the tested isolate, our supernatant broths were adjusted to pH 6.5. All dishes were incubated for 24h at 37^°^C and the inhibition diameter was evaluated by automated colony counter Sphere Flash (IUL, Barcelona). Antibacterial activity was stated for isolates with ZOI measured 10 mm or more.

### Antiviral activity

Cytotoxicity and plaque-reduction tests were conducted against the *SARS-CoV-2* (COVID-19) virus from our biobank to evaluate the antiviral effect of promising isolates. The cytotoxicity effect of tested isolates against viability and proliferation of Vero E6 cells was done by the 3-(4,5-dimethylthiazol-2-yl)−2, 5-diphenyltetrazolium bromide (MTT) method in accord to Mosmann instructions [23] alongside slight alteration [24]. After preparing the stock concentration of the isolates in 10% DMSO, a serial dilution was done in Dulbecco’s Modified Eagle’s Medium (DMEM). Briefly, 100 µl of cell suspension with (3×10^5^ cells/ml) concentration were seeded and incubated for 24h at 37°C with 5% CO_2_. Isolate dilutions were inoculated after 24h in triplicate. Following 24h, the media was aspirated, and cell lines were washed with PBS (phosphate buffer saline) 3 times to be inoculated by 20 µl MTT (5 mg/ml) then incubated for 4h at 37°C followed by medium aspiration. The crystalized formazan formed was solubilized by 200 µl ‎acidic isopropanol (0.04M HCl in pure isopropanol). Formazan solutions' absorbance was obtained using a compatible scanner at wavelength 540 nm, with 620 nm as a standard wavelength. The equation used to calculate the proportion of cytotoxicity versus untreated cells is as follows: [25, 26].

$${\%}\:\text{C}\text{y}\text{t}\text{o}\text{t}\text{o}\text{x}\text{i}\text{c}\text{i}\text{t}\text{y}=\frac{(Absorbance\:of\:untreated\:cells\:-Absorbance\:of\:treated\:cells)}{Absorbance\:of\:untreated\:cells}\times\:100$$

Calculating the cell viability reduced by 50% through plotting the sample concentration versus cytotoxicity percent is called (CC_50_) [27].

The plaque reduction assay was done by seeding a six-well plate by Vero E6 cells (1×10^6^ cells/ml) at 37°C for 24h in 5% CO_2_. 100 µl of the isolates’ safe dilutions (10^−4^, 10^−5^, 10^−6^ and 10^−7^) were mixed by (8×10^4^ PFU/well) plaque-forming unit of diluted COVID-19 then incubated at 37°C for 1h to be inoculated to the cells after aspirating the seeding medium. 1h incubation for virus adsorption was continued by adding 3 ml of supplemented DMEM (with 2% agarose). Solidified Plates were incubated for 3 days at 37°C (till viral plaques formed). After adding 10% formalin for 2h, 0.1% crystal violet was applied before washing. Positive control was assigned for wells infected with untreated virus plus a negative control well without virus. Lastly, the reduction percent in formed plaques compared to control ones was calculated using the following equation after counting the plaques: [25, 26].

$${\%}\:Reduction=\frac{(untreated\:viral\:count-treated\:viral\:count)}{untreated\:viral\:count}\times\:100$$

Using GraphPad Prism® (GraphPad Software Inc., San Diego, CA, USA) version 8, a nonlinear regression study was performed on the percent reduction data and the concentration values that corresponded to them to generate sigmoidal dose-response curves, by which the estimated concentration that would reduce the virus count by 50% (IC50) was derived [27]. The selectivity indices that measure the window between the cytotoxicity and antiviral activity [28] of the tested isolates were calculated according to the following equation: [25, 26].

$$\:Selectivity\:index=\frac{CC50\:of\:isolate\:on\:control\:Vero\:E6\:cells}{\text{I}\text{C}50\:\text{o}\text{f}\:\text{t}\text{h}\text{e}\:\text{i}\text{s}\text{o}\text{l}\text{a}\text{t}\text{e}\:\text{o}\text{n}\:\text{i}\text{n}\text{f}\text{e}\text{c}\text{t}\text{e}\text{d}\:\text{V}\text{e}\text{r}\text{o}\:\text{E}6\:\text{c}\text{e}\text{l}\text{l}\text{s}}$$

### DNA isolation

Using PureLink™ Genomic DNA purification kit (Thermo Fisher Scientific Inc, USA), genomic DNA extraction was done from the isolates’ broth. Per the manufacturer's guidelines, up to (2×10^9^) colonies were harvested after centrifugation and solubilized by Enzymatic Genomic Digestion Buffer. After adding Proteinase K, incubation at 55°C till cell lysis was done (30 min to 4h). After that, RNase A was added and mixed properly after incubation at room temperature for 2 minutes with Genomic Lysis/Binding Buffer. The whole content was mixed well with 100% ethanol and then washed twice by buffer AW1 and AW2 respectively. The eluted DNA was quantified for its concentration and purity by a previously calibrated Qubit™ 2.0 fluorometer instrument (Invitrogen, United States) with DNA HS assay kit and Nanodrop 8000 (Thermo Fisher Scientific, Waltham, MA, USA) as directed by the manufacturer, then kept at −20°C till used.

### Genome sequencing

DNA libraries were prepared using the Nextera-XT DNA Library Prep kit (Illumina, San Diego, CA) following the manufacturer's guidelines with an initial DNA concentration of 1 ng/µl. By adding adapters of (i7) and (i5) indexes, Nextera transposome was used to fragment and tagment gDNA of tested samples. Amplified libraries were purified using single-sided Agencourt AMPure XP beads, normalized and diluted. Based on sample complexity, more than three samples might be pooled by run and then sequenced with the Illumina MiSeq 300-cycle cartridge kit for cluster generation on the Miseq Platform (Illumina, San Diego, CA, United States) at Genetics Research Unit, Main Chemical Warfare Laboratories, Ministry of Defense, Egypt.

### Genome features, assembly and annotation

After the run had finished, primary analysis of the sequencing run parameters was done on board. FastQC (version 0.12.1) was applied to the raw reads [29]. Assembly quality study was processed by the comprehensive genome analyses tool at the PATRIC (PathoSystems Resource Integration Center) platform [30]. The obtained reads from previous analysis using FastQC and QUAST with good quality were annotated using RASTtk (Rapid Annotation using Subsystem Technology tool kit) [31].

### Phylogenomic analysis

To identify the representative genomes and closest reference, Mash/MinHash was used [32]. From these genomes, PGFams (PATRIC global protein families) were chosen to ascertain the phylogenetic position [33]. These families' protein sequences were aligned using MUSCLE (Multiple Sequence Alignment) [34], and each sequence's nucleotides were allocated to the protein alignments. After concatenating the combined set of nucleotide and amino acid alignments into a data matrix, RaxML (Randomized Axelerated Maximum Likelihood) [35] was utilized to quickly bootstrap this matrix for analysis [36].

### Screening of functional genes and genome stability

The cluster genes correlated to the probiotic properties were detected using (antiSMASH 7.0) [37]. The antibiotic resistance genes were predicted by ARTS 2.0 (Antibiotic Resistant Target Seeker) [38], AMR genes discovery tool based on k-mer in PATRIC and ResFinder software (version 4.5.0) [39]. The Bacteriocin Database BAGEL4 was applied to analyze the arrangement of the bacteriocin-encoding genes [40]. Presumed mobilome and phages, were searched under PhaBOX [41]. The CRISPR (clustered regularly interspaced short palindromic repeats) and CRISPR/Cas (CRISPR associated genes) were identified using the CRISPRCasFinder [42].

### Statistical analysis

Standard deviation was estimated for each characterization experiment, which was done out in triplets. GraphPad Prism 6.0 was also used for statistical analysis. A p-value of less than 0.05 was deemed statistically significant.

## Results

### Identification of isolates and screening of the probiotic properties

Out of nineteen isolated samples, 15 samples gave identification on MRS selective media as white-smooth-mucoid rod-shaped colonies, small-white-smooth long chains of cocci and circular-white-smooth opaque colonies with entire margins and convex elevation existing in pairs respectively and gram-positive staining when has been applied for microscopic examination. Biochemical confirmation by the VITEK® 2C automated platform revealed five isolates as *lactobacillus delbrueckii, Streptococcus thermophilus* and *Pediococcus acidilactici* with excellent identification and was screened for the following probiotic criteria.

### Tolerance to sodium chloride (NaCl)

Five LAB isolates were tested for their tolerance to different NaCl concentrations, out of which three tolerants only developed properly at doses of 3%, 4%, and 5%, while slow growth was noticed at concentrations of 6% and 7% NaCl. At 7% NaCl dose, the growth slowed greatly after both 2 and 5h of incubation (Table 1).

**Table 1:** Tolerance of isolated samples for NaCl concentrations at 2h and 5h

Isolate	NaCl 3%		NaCl 4%		NaCl 5%		NaCl 6%		NaCl 7%	
	**Count**	**Abs**	**Count**	**Abs**	**Count**	**Abs**	**Count**	**Abs**	**Count**	**Abs**
G2-2h	1299.32	0.718	1728.99	0.701	1115.10	0.599	998.20	0.457	400.78	0.152
R1-2h	1391.25	0.802	1604.58	0.784	1145.69	0.599	1004.09	0.501	166.99	0.199
S6-2h	1199.36	0.756	1254.98	0.704	1028.41	0.696	899.99	0.498	499.74	0.204
M3-2h	894.36	0.7	800.15	0.541	699.81	0.497	581.06	0.248	274.30	0.201
M7-2h	647.21	0.578	287.95	0.365	200.36	0.331	301.85	0.255	114.88	0.199
G2-5h	1287.02	0.655	1012.36	0.587	1100.73	0.491	897.05	0.301	336.18	0.174
R1-5h	1313.21	0.719	1148.69	0.678	1079.16	0.52	907.55	0.478	128.77	0.182
S6-5h	998.78	0.697	990.48	0.58	879.72	0.412	758.11	0.369	225.88	0.147
M3-5h	827.69	0.581	697.48	0.502	492.02	0.481	428.49	0.28	332.85	0.183
M7-5h	950.48	0.5	587.20	0.412	546.78	0.385	500.33	0.281	178.99	0.101

Count: the total number of colonies were summed after exposure to stress conditions on agar plates.

Abs: refer to the absorbance of the growing colonies in the broth measured at OD_600._

### Acid tolerance

The three isolates that grew well at 5% NaCl concentration survived at low pH and showed resistance to acid stress. The resistant strains could survive at pH 4 (tolerance > 50%). After 5 h at pH 4, R1 had the highest tolerance (76.96%) and S6 had the lowest tolerance (68.86%) (Table 2). At pH 3, no tolerant strains were detected (resistance percentage < 50%).

**Table 2:** Acid tolerance of isolated samples at 2h and 5h

Isolate	pH 3		pH 4		pH 5		pH 6		pH 7		resistance % at pH 4
	**Count**	**Abs**	**Count**	**Abs**	**Count**	**Abs**	**Count**	**Abs**	**Count**	**Abs**	
G2-2h	129.90	0.402	101.96	0.564	719.43	0.801	501.63	0.698	917.19	0.908	62.11
R1-2h	78.66	0.297	116.36	0.498	616.36	0.598	689.08	0.697	1005.85	0.849	58.65
S6-2h	45.36	0.398	99.41	0.588	878.39	0.602	645.88	0.744	908.39	0.904	65.04
M3-2h	82.37	0.195	78.97	0.195	377.95	0.298	801.80	0.569	1045.48	0.861	22.64
M7-2h	97.59	0.169	614.41	0.217	228.22	0.321	369.76	0.666	972.88	0.802	27.06
G2-5h	75.85	0.401	214.75	0.591	1099.66	0.805	908.83	0.695	1111.76	0.84	70.35
R1-5h	125.70	0.367	45.34	0.698	980.75	0.746	1005.64	0.791	1173.39	0.907	76.96
S6-5h	105.08	0.457	298.31	0.648	874.34	0.87	905.51	0.787	1024.56	0.941	68.86
M3-5h	62.54	0.209	205.25	0.402	139.41	0.399	408.81	0.47	965.03	0.875	45.94
M7-5h	104.49	0.345	359.15	0.408	241.53	0.409	621.69	0.518	602.12	0.901	45.28

Count: the total number of colonies were summed after exposure to stress conditions on agar plates

Abs: refer to the absorbance of the growing colonies in the broth measured at OD_600_.

### Bile tolerance

**Fig. 1:**
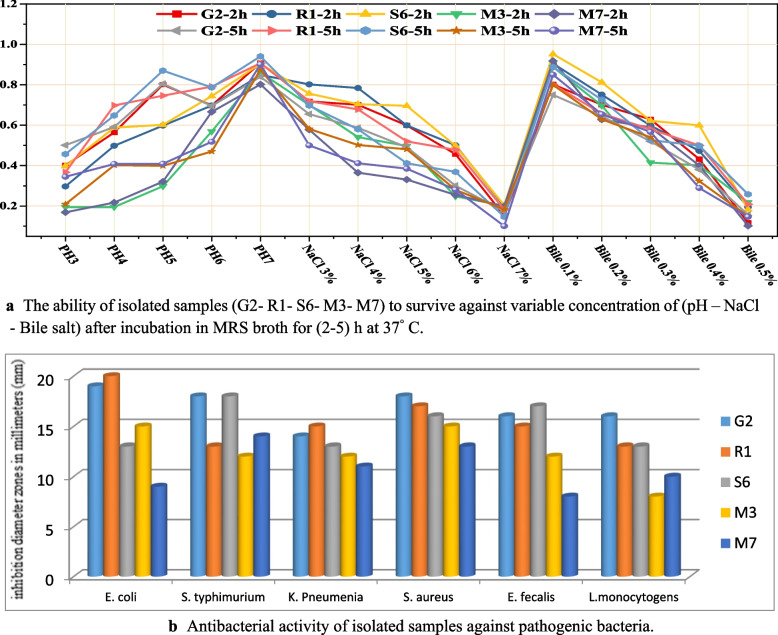
**a** The ability of isolated samples (G2- R1- S6- M3- M7) to survive against variable concentration of (pH – NaCl - Bile salt) after incubation in MRS broth for (2–5) h at 37° C. **b** Antibacterial activity of isolated samples against pathogenic bacteria

Inhibition was observed by increasing bile salt concentrations, but the three isolates tested continued to grow with increasing incubation time (resistance > 50%) but could not withstand bile salt concentrations of 0.5%. The highest resistance was reported for R1 (62.34%) and the lowest for G2 (50.66%), with 0.4% at 5h (Table 3). However, no isolates were resistant (percent resistance < 50%) in the 0.5% range. The challenges promising isolates face to survive these stressful conditions, which resemble those of the stomach and human intestine, are shown in Fig. 1a.

**Table 3:** Bile tolerance of isolated samples at 2h and 5h

Isolate	Bile 0.1%		Bile 0.2%		Bile 0.3%		Bile 0.4%		Bile 0.5%		resistance at 0.4%
	**Count**	**Abs**	**Count**	**Abs**	**Count**	**Abs**	**Count**	**Abs**	**Count**	**Abs**	
G2-2h	1245.95	0.802	1099.15	0.701	952.77	0.629	699.47	0.432	321.88	0.118	53.87
R1-2h	1208.41	0.905	999.58	0.751	852.95	0.598	701.71	0.473	254.20	0.195	52.27
S6-2h	1145.63	0.952	891.2	0.812	748.99	0.622	550.41	0.599	410.25	0.183	62.92
M3-2h	951.77	0.888	820.36	0.697	648.12	0.415	412.88	0.401	302.12	0.218	45.16
M7-2h	1025.12	0.918	920.15	0.628	752.66	0.589	441.51	0.4	208.94	0.102	43.57
G2-5h	1045.75	0.75	854.45	0.645	781.66	0.52	612.4	0.38	178.55	0.156	50.66
R1-5h	1104.95	0.802	942.77	0.657	719.87	0.58	599.85	0.5	199.71	0.209	62.34
S6-5h	999.47	0.888	802.72	0.726	651.88	0.526	499.22	0.499	194.02	0.258	56.19
M3-5h	901.88	0.799	793.69	0.629	570	0.54	369.85	0.323	169.77	0.149	40.43
M7-5h	988.92	0.85	796.77	0.654	506.11	0.567	409.88	0.289	102.88	0.147	34

Count: the total number of colonies were summed after exposure to stress conditions on agar plates.

Abs: refer to the absorbance of the growing colonies in the broth measured at OD_600_.

### Antibiotics susceptibility

We investigated our trials for antibiotic resistance on MHA plates by disc diffusing technique and the results are presented in Table 4. Three isolates showed resistance to stress factors, and were highly sensitive to azithromycin, chloramphenicol, ciprofloxacin, erythromycin, clarithromycin, nalidixic acid and neomycin while sensitive to ampicillin, tetracycline, streptomycin, and gentamicin. Interestingly, these isolates share less sensitivity towards penicillin, trimethoprim, sulfamethizole and ceftriaxone. It would be advantageous if probiotic microorganisms could possess antibiotic resistance to survive antibiotic treatment in the gastrointestinal tract.

**Table 4:** Antibiotic susceptibility profile of isolated samples with MHA after 24 h

	G2	R1	S6	M3	M7
Ampicillin	++	++	+++	+	R
Azithromycin	++++	++++	+++	++	++++
Penicillin	++	+	++	R	+
Chloramphenicol	+++	++++	+++	+	++
Ciprofloxacin	++++	++++	++	++++	++
Clarithromycin	+++	++++	+++	++	++++
Erythromycin	+++	++	+++	R	++
Tetracycline	++	+	++	++	R
Nalidixic acid	++++	+++	+++	+++	+++
Trimethoprim	R	++	+	R	+
Ceftriaxone	+	++	+	+++	+++
Streptomycin	+	+++	++	+++	+
Gentamicin	+	+	R	+	+
Neomycin	++++	++++	++++	++++	++++
Sulfamethizole	R	+	+++	+	R

R = resistant, +=less sensitive, ++=moderately sensitive, +++=sensitive, ++++=highly sensitive

### Antibacterial activity

Six pathogens were investigated to determine the antibacterial effects of the evaluated isolates (Fig. 1b). The bacterial cell-free fluids tested showed inhibition against the pathogenic strains examined. *S. aureus* was an extremely sensitive pathogen to all the tested isolates. Isolate G2 was the most potent isolate as in (Table 5).

**Table 5:** Zone of inhibition of Antibacterial activity against pathogenic bacteria

	G2	R1	S6	M3	M7
*E. coli*	19	20	13	15	9
*S. typhimurium*	18	13	18	12	14
*K. Pneumenia*	14	15	13	12	11
*S. aureus*	18	17	16	15	13
*E. fecalis*	16	15	17	12	8
*L.monocytogens*	16	13	13	8	10

### Antiviral activity

Since G2, S6, and R1 isolates demonstrated promising probiotic potential, further investigations were focused on these strains. The cytotoxicity of different concentrations of tested isolates (G2-R1-S6) on the viability of the Vero E6 cell line for 24 h, showed that 10^−4^, 10^−5^, 10^−6^ and 10^−7^ were safe. These concentrations were subsequently tested for their antiviral effects against SARS-CoV-2.". The findings revealed an inhibition percentage as shown in Table 6. Isolate G2 was the active inhibitory one.

**Table 6:** Antiviral activity of effective antibacterial isolates against *SARS-CoV-2*

Sample	Conc (CFU/ml)	Cytotoxicity %	Initial Viral count (PFU/ml)	Post Viral count (PFU/ml)	Inhibition %
G2	10^−4^	72.51	8*10^4^	2.42*10^4^	69
	10^−5^	69.74		4.91*10^4^	38
	10^−6^	54.13		5.62*10^4^	29
	10^−7^	44.99		7.41*10^4^	7
R1	10^−4^	71.36	8*10^4^	2.99*10^4^	62
	10^−5^	58.89		4.52*10^4^	43
	10^−6^	51.92		5.14*10^4^	35
	10^−7^	47.97		7.05*10^4^	11
S6	10^−4^	68.12	8*10^4^	3.02*10^4^	62
	10^−5^	61.41		4.23*10^4^	47
	10^−6^	50.85		5.66*10^4^	29
	10^−7^	48.42		7.39*10^4^	7

### Genome sequencing

We obtained a complete genome sequence of three promising strains. A sequencing run was carried out on the Miseq platform for samples tested and identified during the study by the Nextera XT library preparation kit. The obtained data underwent a system analysis that resulted in 1350 k/mm2 total clusters, 90.8% a cluster PF, 96.5% Q30 (Read 1) and 97.5% Q30 (Read 2).

### Genome features, assembly, annotation

The raw reads gave a good quality score on FastQC quality control checks on raw sequence data. According to the obtained data, sample no.1 coded by (G2) was assembled and resulting in 422 contigs with a genome length of 2,036,424 bp, With a typical G + C composition of 49.37%. The smallest sequence segment at 50% of the genome, which is known as N50 length, is 16,897 bp. The shortest number of contigs whose length sum produces N50 which defines the L50 count, is 33. Sample (G2) had been annotated with RASTtk (RAST tool kit) and given a distinct taxonomic classification as: cellular organisms > Bacteria > Terrabacteria > Firmicutes > Bacilli > Lactobacillales > Lactobacillaceae. This genome has 2,381 CDS (protein coding sequences), 61 tRNA (transfer RNA) genes, and 4 rRNA (ribosomal RNA) genes. The annotation involved 729 hypothetical proteins and 1,652 proteins having functional assignments out of which 559 EC numbers (proteins have Enzyme Commission), 463 GO assignments (proteins have Gene Ontology), and 388 proteins that were mapped to KEGG pathways and 2,232 proteins that belong to the PGFams (cross-genus protein families).

All these genomic features of our sequenced samples coded by (G2, R1 and S6) are listed in (Table 7). Samples coded by (G2 and S6) were assembled and resulted (1,016 and 19) contigs having (3,945,522 and 1,998,717) bp of genomic length of, with atypical GC content of (44.27 and 42.01) % respectively. The N50 length is (15,882 and 313,402) bp and the L50 count is (49 and 3) in the same respect. The annotation of sample (R1) revealed the taxonomy belongs to the family *Streptococcaceae*, while sample (S6) belongs to *Lactobacillaceae*. Both genomes have in order (4,902 and 1,963) CDS, (8 and 3) rRNA genes, and (89 and 15) tRNA genes. The annotation comprises (1,414 and 456) hypothetical proteins and (3,488 and 1,507) proteins have functional assignments divided into (1,225 and 547) EC proteins, (1,034 and 456) with GO assignments, and (866 and 387) proteins that had been mapped to KEGG pathways and (4,591 and 1,922) PGFams proteins.

**Table 7:** Assembly and annotation genomic features of the sequenced isolates

Isolate (Job ID)	G2 (2249246)	R1 (1799138)	S6 (9343)
Source	yogurt	yogurt	sour milk
**Assembly details**:			
Contigs	422	1,016	19
GC Content	49.37	44.27	42.01
Contig L50	33	49	3
Genome Length (bp)	2,036,424	3,945,522	1,998,717
Contig N50	16,897	15,882	313,402
Total genes	1954	3935	1914
Core/ Essential genes	549	732	635
**Annotated Genome Features**:			
CDS	2,381	4,902	1,963
Repeat Regions	141	227	50
tRNA	61	89	15
rRNA	4	8	3
**Protein Features**:			
Hypothetical proteins	729	1,414	456
Proteins with functional assignments	1,652	3,488	1,507
Proteins with EC number assignments	559	1,225	547
Proteins with GO assignments	463	1,034	456
Proteins with Pathway assignments	388	866	387
Proteins with PATRIC (PGfam)	2,232	4,591	1,922

**Fig. 2:**
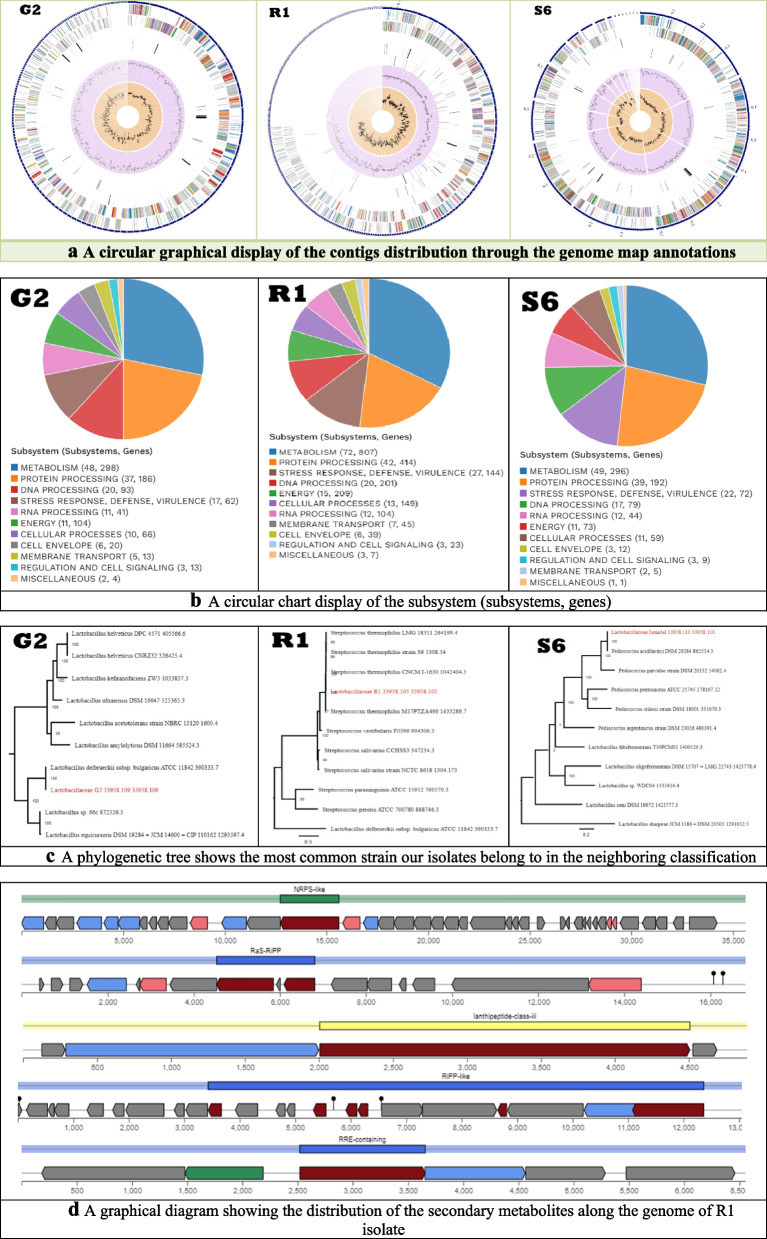
**a** A circular graphical display of the contigs distribution through the genome map annotations. **b** A circular chart display of the subsystem (subsystems, genes). **c** A phylogenetic tree shows the most common strain our isolates belong to in the neighboring classification. **d** A graphical diagram showing the distribution of the secondary metabolites along the genome of R1 isolate

The distribution of the genome annotations is shown in a circular graph (Fig. 2a). From outer to inner rings, this comprises the contigs, CDS on the forward strand, CDS on the reverse strand, RNA genes, CDS with homology to known antimicrobial resistance genes, CDS with homology to known virulence factors, GC content and GC skew. The subsystem to which these genes belong is indicated by the colors of the CDS on the forward and reverse strands (Fig. 2a).

Subsystems are collections of proteins that work together to carry out a particular biological function or structure complex. Examination of subsystems unique to each genome is part of the PATRIC annotation process. (Fig. 2b) gives a summary of the subsystem values for these genomes. The most represented subsystem features are metabolism, protein processing, defense, stress response, energy, virulence, cellular processes, DNA processing, RNA processing, cell envelope, membrane transport, miscellaneous, regulation and cell signaling.

### Phylogenetic tree analysis

Our strains G2, R1 and S6 showed maximum sequence similarity with *Lactobacillus delbrueckii* subsp. *bulgaricus, Streptococcus thermophiles*, and *Pediococcus acidilactici*, respectively. Based on the complete genomic sequence, a phylogenetic tree was created after concatenating the combined set of nucleotide and amino acid alignments into a data matrix, and RaxML was utilized to resolve this matrix to understand the phylogenetic relationship among LAB strains as shown in (Fig. 2c).

### Bioinformatics analysis

Numerous annotated genes are homologous to known drug targets, transporters, and antibiotic-resistant genes. Antibiotic resistance phenotype is not always implied by the existence of AMR-related genes, even full ones, in a particular genome. This was confirmed through the ResFinder software (version 4.5.0), which proved that the samples were free of any resistant phenotypes. It is crucial to consider certain AMR pathways, particularly the existence or lack of SNP mutations that indicate resistance. Table 8 provides an overview of the AMR genes identified in this genome as the relevant AMR pathway.

**Table 8:** Specialty and AMR genes of the sequenced isolates

Strain (Job ID)	G2 (2249246)	R1 (1799138)	S6 (9343)
Specialty Genes:			
Antibiotic Resistance	19	45	22
Drug Target	5	12	1
Transporter	5	35	4
**Antimicrobial Resistance Genes**:			
Antibiotic target in susceptible species	Alr, Ddl, EF-G, EF-Tu, folA, Dfr, folP, gyrA, gyrB, inhA, fabI, Iso-tRNA, kasA, MurA, rpoB, rpoC, S10p, S12p	Alr, Ddl, EF-G, EF-Tu, folA, Dfr, folP, gyrA, gyrB, inhA, fabI, Iso-tRNA, kasA, MurA, rpoB, rpoC, S10p, S12p	Alr, Ddl, EF-G, EF-Tu, folA, Dfr, folP, gyrA, gyrB, inhA, fabI, Iso-tRNA, kasA, MurA, rho, rpoB, rpoC, S10p, S12p
Antibiotic target modifying enzyme	--	RlmA(II)	RlmA(II)
Antibiotic target replacement protein	--	FabK	FabV
Gene conferring resistance via absence	gidB	gidB	gidB
Protein altering cell wall charge conferring antibiotic resistance	GdpD, PgsA	GdpD, MprF, PgsA	GdpD, PgsA
Regulator modulating expression of antibiotic resistance genes	--	LiaF, LiaR, LiaS	--

AntiSMASH enables quick discovery, annotation, and investigation of gene clusters involved in secondary metabolite production throughout the whole genome of bacteria. It identified the secondary metabolite regions (especially biosynthetic genes) that exist on different contigs along the genome of the analyzed isolates as shown in (Table 9).

**Table 9:** Biosynthetic secondary metabolites tracked by AntiSMASH software

	G2	R1	S6
RRE-containing	contig_225 - Region 1	contig_160 - Region 1	--
		contig_522 - Region 1	
RiPP-like	contig_107 - Region 1	contig_71 - Region 1	--
		contig_278 - Region 1	
NRPS-like	--	contig_2 - Region 1	--
RaS-RiPP	--	contig_15 - Region 1	--
		contig_24 - Region 1	
lanthipeptide-class-i	--	contig_22 - Region 1	--
lanthipeptide-class-iii	contig_95 - Region 1	--	--

The ARTS software confirmed all the resulting analyses obtained from AntiSMASH related to core/essential genes. Bacteriocin database BAGEL4 revealed the arrangement of the bacteriocin-encoding genes for G2 and R1 isolate as listed in Table 10, while no hits were found for S6 isolate.

**Table 10:** Bacteriocin-encoding genes localized on assembly contigs tracked by BAGEL4 software

	G2	R1	S6
70.3; Helveticin-J	assembly_contig_136.113.AOI_01	assembly_contig_293.266.AOI_01	--
64.3; Enterolysin_A	assembly_contig_32.21.AOI_01	--	--
231.2; BlpD	--	assembly_contig_71.184.AOI_01	
295.1; Streptide	--	assembly_contig_15.190.AOI_01	
Sactipeptides	--	assembly_contig_15.190.AOI_01	--
183.1; FlvA2h	--	assembly_contig_108.51.AOI_01	--
lanthipeptide-class-i	--	assembly_contig_22.4.AOI_01	--
Lanthipeptide_class_IV	assembly_contig_95.51.AOI_01	assembly_contig_202.70.AOI_01	--

**Fig. 3:**
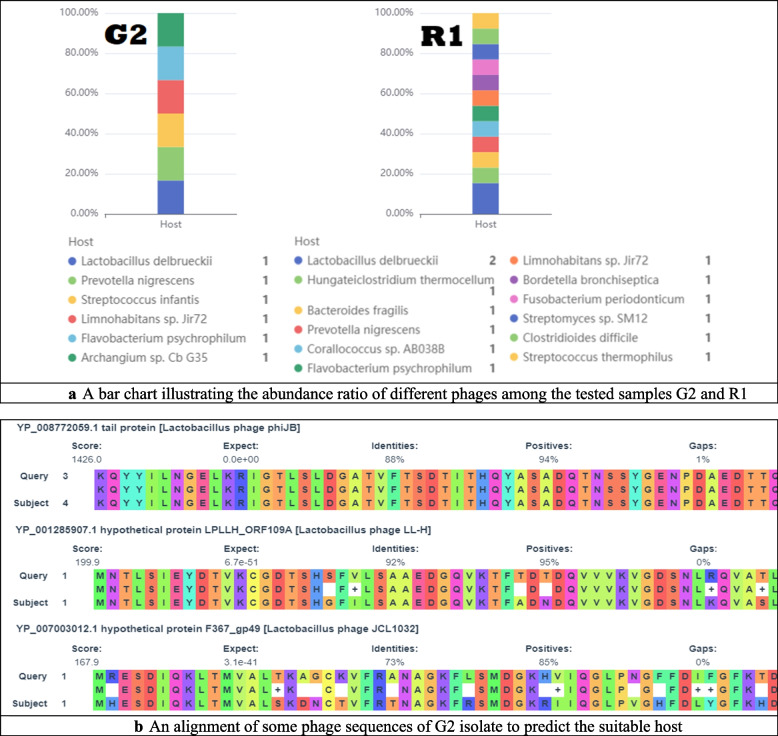
**a** A bar chart illustrating the abundance ratio of different phages among the tested samples G2 and R1. **b** An alignment of some phage sequences of G2 isolate to predict the suitable host

Prophages and other insertion sequences which are named mobile genetic elements, were identified in the tested isolates by PhaBOX online tool. Two isolates were found to have phage sequences either virulent or temperate. Isolate G2 has six phage sequences out of which, one phage belongs to *Peduoviridae* family that matched *Prevotella nigrescens* as a suitable host. Isolate R1 showed thirteen phages, two of which belong to (*Peduoviridae* as isolate G2 and *Ackermannviridae* that matched *Clostridioides difficile* as suitable host). In Fig. 3a and b we found that the rest of the found phages have unknown families but matched a suitable host as shown in Tables 11 and 12. Isolate S6 doesn’t have any phage sequences.

**Table 11:** Phage detected contigs in G2 isolate by PhaBOX

ID	Contig no.	Length	Lifestyle	Family	Host	Type
1	assembly_contig_8	37325	virulent	unknown	*Lactobacillus delbrueckii*	CRISPR
2	assembly_contig_26	20947	virulent	*Peduoviridae*	*Prevotella nigrescens*	CRISPR
3	assembly_contig_83	5784	temperate	unknown	*Streptococcus infantis*	CRISPR
4	assembly_contig_89	5464	virulent	unknown	*Limnohabitans sp. Jir72*	CRISPR
5	assembly_contig_93	5150	virulent	unknown	*Flavobacterium psychrophilum*	CRISPR
6	assembly_contig_134	3239	virulent	unknown	*Archangium sp. Cb G35*	CRISPR

**Table 12:** Phage detected contigs in R1 isolate by PhaBOX

ID	Contig no.	Length	Lifestyle	Family	Host	Type
1	assembly_contig_7	37288	virulent	unknown	*Lactobacillus delbrueckii*	CRISPR
2	assembly_contig_21	26110	temperate	unknown	*Hungateiclostridium thermocellum*	CRISPR
3	assembly_contig_75	12400	temperate	unknown	*Bacteroides fragilis*	CRISPR
4	assembly_contig_94	10664	temperate	*Peduoviridae*	*Prevotella nigrescens*	CRISPR
5	assembly_contig_182	5537	temperate	unknown	*Corallococcus sp. AB038B*	CRISPR
6	assembly_contig_191	5150	virulent	unknown	*Flavobacterium psychrophilum*	CRISPR
7	assembly_contig_197	5002	virulent	unknown	*Limnohabitans sp. Jir72*	CRISPR
8	assembly_contig_217	4549	temperate	unknown	*Bordetella bronchiseptica*	CRISPR
9	assembly_contig_248	3968	virulent	unknown	*Fusobacterium periodonticum*	CRISPR
10	assembly_contig_253	3864	virulent	unknown	*Streptomyces sp. SM12*	CRISPR
11	assembly_contig_262	3704	virulent	unknown	*Lactobacillus delbrueckii*	CRISPR
12	assembly_contig_269	3572	temperate	*Ackermannviridae*	*Clostridioides difficile*	CRISPR
13	assembly_contig_300	3002	virulent	unknown	*Streptococcus thermophilus*	CRISPR

All isolates were searched for presumed CRISPR-Cas coding sequences. All three isolates showed these repeated sequences that contain one or more associated Cas-gene or consensus repeats and the spacer genes as shown in the following tables. They have (5, 11, 2) CRISPR sequences respectively, associated with (2, 7, 3) Cas in the same order and differentiated to (8, 25, 7) clusters in the arrange (G2, R1, S6) as in Tables 13, 14, and 15.

**Table 13:** CRISPR/Cas clusters detected in G2 isolate by CRISPRCasFinder software

Genome location	Length (bp)	CRISPR	Spacer	Cas	genes	Cas clusters
contig_12	32426	1	1	0	0	---
contig_33	15882	1	21	2	8	Cas10_0_IIIA, Cas2_0_I-II-III, Cas6_0_I-III, Csm2_0_IIIA, Csm3_0_IIIA, Csm3_0_IIID, Csm4_0_IIIA, Cas1_0_II
contig_118	3812	1	61	0	0	---
contig_173	1959	1	31	0	0	---
contig_237	970	1	14	0	0	---

**Table 14:** CRISPR/Cas clusters detected in R1 isolate by CRISPRCasFinder software

Genome location	Length (bp)	CRISPR	Spacer	Cas	genes	Cas clusters
contig_13	32463	1	18	2	4	Cas9_1_II, Csn2_0_IIA, Cas1_0_II, Cas2_0_I-II-III
contig_48	16068	1	4	1	9	Cas10_0_IIIA, Cas1_0_I-II-III, Cas2_0_I-II-III-V, Cas6_0_I-III, Csm2_0_IIIA, Csm3_0_IIIA, Csm4_0_IIIA, Csm5_0_IIIA, Csm6_0_IIIA
contig_49	15882	1	21	2	8	Cas10_0_IIIA, Cas2_0_I-II-III, Cas6_0_I-III, Csm2_0_IIIA, Csm3_0_IIIA, Csm3_0_IIID, Csm4_0_IIIA, Cas1_0_II
contig_158	6686	1	1	2	4	Cas9_0_II, Csn2_0_IIA, Cas1_0_II, Cas2_0_I-II-III
contig_214	4588	1	1	0	0	---
contig_252	3888	1	61	0	0	---
contig_322	2756	1	37	0	0	---
contig_401	1967	1	25	0	0	---
contig_408	1890	1	30	0	0	---
contig_538	1183	1	18	0	0	---
contig_1071	425	1	3	0	0	---

**Table 15:** CRISPR/Cas clusters detected in S6 isolate by CRISPRCasFinder software

Genome location	Length (bp)	CRISPR	Spacer	Cas	genes	Cas clusters
contig_2	315707	1	13	2	5	Cas2_0_I-II-III, Cas3_0_I, Cas9_0_II, Csn2_0_IIA, Cas1_0_II
contig_7	116484	1	1	1	2	Cas3_0_I, Cas3_0_I

## Discussion

Probiotics are defined by the World Health Organization (WHO) as "living microbes that, when administered in adequate amounts, confer a health benefit on the host." Many food products available today contain live microorganisms added to promote general health benefits. Worldwide, probiotics are employed in a variety of products such as medicines, animal feed, and foods. In Egypt, all probiotics used in dietary supplements available on the medical market are imported. It became necessary to identify and describe a potential local probiotic strain for use in foods and medicines. The *in vitro* selection criteria for probiotics include bile and acid resistance, which enable these microorganisms to survive and thrive in the GIT (gastrointestinal tract) [43].

Nineteen samples from various Egyptian marketplaces, both natural and commercial, were collected and cultured on MRS agar. Among these, five isolates G2, R1, S6, M3 and M7 were confirmed with 99% excellent confirmation by VITEK system as *Lactobacillus delbrueckii* for G2, M3 and M7 isolates, *Streptococcus thermophilus* for R1 isolate and *Pediococcus acidilactici* for S6 isolate. These isolates subsequently underwent characterization for probiotic properties. Out of these five isolates, three isolates, G2, R1, S6 exhibited strong tolerance to 5% NaCl after 5h and survive well at pH 4 (with tolerance > 50%). Highest endurance was recognized in R1 (76.96%) while S6 showed the lowest (68.86%) at pH 4 after 5h (Table 2). However, none of the isolates exhibited tolerance at pH 3, as all had tolerance levels below 50%. Acid resilience is crucial for probiotic strains to survive the initial gastric shock in the stomach, where pH typically ranges between 2 and 3 [13].

Idoui [44] reported the persistence of *L. plantarum* BJ0021 at 3.0 pH and proved that, in contrast to isolated *Lactobacillus* from human gastrointestinal tracts, strains of *L. fermentum, L. delbrueckii* subsp. *bulgaricus*, and *L. gasseri* had greater acid endurance. Furthermore, acid-resistant isolates developed and persisted at a bile concentration of 0.4% for 5h. The highly resistant one was R1 (62.34%) and the lowest was G2 (50.66%) at 0.4% after 5h (Table 3). None of the isolates demonstrated tolerance at 0.5% bile, as all exhibited resistance below 50%. This aligned with the proof that the probiotic isolates were resistant to pH 3 and 0.3% bile [18]. As notice by Gilliland [45], lactobacilli isolated from animal intestines showed a greater resistance to bile salts when compared to those isolated from dairy products. showed. *Streptococcus thermophilus and Pediococcus acidilactici* under investigation are of dairy origin and are being assessed for their probiotic characteristics. Garriga et al. [46] revealed that chosen LAB strains are tolerant to 4% bile salt. LAB resistance varies due to the presence of bile salt hydrolase, an enzyme that conjugates bile to reduce its toxic effects [47].

One suitable criterion for selecting a probiotic culture is antibiotic sensitivity [48]. Using the agar disc diffusion method, the antibiotic sensitivity of all tested isolates was determined. Our selected isolates exhibited resistance to several antibiotics, consistent with findings by Botes et al. [49] who reported that *L. casei* was suppressed by various commercial antibiotics. Generally, *Lactobacilli* are susceptible to penicillin and β-lactamase targeting the cell wall but refractory to cephalosporins [50]. The bulk of LAB strains seem to be less inhibited by most inhibitors against nucleic acid synthesis. *Lactobacilli*, however, were often sensitive to low doses of many inhibitors of protein synthesis, like tetracycline and chloramphenicol, because of the existence of its resisting genes, which were occasionally detected in combination with another [51]. As antibiotic resistance among LAB is advantageous to survive in the GIT during antibiotic courses (REF), our resistant isolates to stress factors were highly sensitive to azithromycin, chloramphenicol, ciprofloxacin, erythromycin, clarithromycin, nalidixic acid and neomycin while sensitive to ampicillin, tetracycline, streptomycin, and gentamicin. Interestingly, these isolates share a common less sensitivity towards penicillin, trimethoprim, sulfamethizole and ceftriaxone.

Since probiotic bacteria regulate pathogenic species in the digestive system and boost immunity conferring a vital role to human health [52], antibacterial action is among probiotics' principal characteristics. Thus, the chosen isolates were examined against six pathogens as shown in Fig. 1b. All bacterial cell-free fluids tested showed inhibition against the pathogenic strains examined. *S. aureus* was an extremely sensitive pathogen to all the tested isolates. Isolate G2 was the most potent isolate. Tulumoglu and associates introduced the antibacterial efficacy of probiotics against *S. aureus*, *P. aeruginosa*, and *E. coli* [48]. PH values, struggle for nutrients, or the generation of compounds with a bacteriostatic or bactericidal effect, such as bacteriocin and bacteriocin-like compounds, may be related to probiotic strains' antibacterial activity [53], plus the antimicrobial metabolites [54], like acetic acid or lactic acid, carbon dioxide, diacetyl, aldehydes, hydrogen peroxide and other metabolites that serve as suppression substances for harmful bacteria [55].

Many previous studies stated that probiotics have good results in treating Covid-19 [56], even in high mutation rates [57]. As predicted, depending on the recent findings of probiotics effect in elimination of covid-19 virus [25], the antiviral activity toward *SARS-CoV-2* after safe viability testing on Vero E6 cell-line for 24h was demonstrated by the successful antimicrobial isolates. The results in Table 6 revealed an inhibition percentage for G2 isolate which was the active inhibitory one.

The promising isolates G2, R1 and S6 exhibiting resistance against acid pH, bile salt and some antibiotics were picked for further research due to antibacterial and antiviral action. These isolates, derived from milk, were studied for probiotic qualities and investigated for safety and efficacy for human uses. All isolates were subjected to high-throughput complete genome next generation sequencing and analyzed for its functional characterization. Although this approach is still costly, WGS technology has become much less expensive over the past ten years, and this trend will continue when new systems are developed [58].

DNA next-generation sequencing has rapidly developed into an extensive technique for material identification and analysis. The WGS resulting data was analyzed using several pipelines, outlined in the methodology. This approach can be customized to provide the required taxonomic accuracy, for example, strain-scale microorganism identification [59]. Overall, high-throughput sequencing provided amazing information that summed up the significance of the strains that were evaluated. WGS was done for G2, R1 and S6 isolates with excellent parameters resulting from onboard primary analysis with 1350 k/mm2 total clusters, 90.8% a cluster PF and 97% total Q30, then the raw reads underwent further check and gave a good quality score on FastQC tool.

After assembly, all reads were annotated revealing 422 contigs with 2.04 Mbp genome length and 49.37% GC content for the G2 isolate, 1016 contigs with 3.95 Mbp genome length and 44.27% GC content for the R1 isolate, and 19 contigs with 1.99 Mbp genome length and 42.01% GC content for S6 isolate. Omic-based techniques and genetic sequencing can effectively identify probiotic-related genes, including those linked to metabolism, genomic adaptability, stability, antimicrobial tolerance, virulence, and safety In addition, *in vivo*, *in vitro*, and omic research are essential tools for assessing probiotic abilities, including tolerance to acidic environments, bile acids, antimicrobial substances, immuno-modulation, and adhesive mechanisms [60]. Probiotics benefit recipients' health by modifying their gut microbiota, therefore limiting pathogen colonization, regulating the host immunity response, reducing serum cholesterol, exhibiting antidiabetic, antihypertensive and antioxidant actions, and creating bacteriocins [61]. Phylogenetic analysis revealed that our isolates are closely related to *Lactobacillus delbrueckii* subsp. *bulgaricus, Streptococcus thermophilus* and *Pediococcus acidilactici.* Phylogenetic trees were created after the combined set of nucleotide and protein alignments were placed into a concatenated matrix and analyzed by PATRIC and RaxML as a part of comprehensive genome analysis. All these data were deposited in the NCBI database.

The genome subsystem analysis, represented in Fig. 2b, includes protein processing, metabolism, stress response, defense, virulence, DNA processing, cellular processes, energy, RNA processing, membrane transport, cell envelope, miscellaneous, regulation and cell signaling. In our study, the genome was analyzed for the screening of antimicrobial tolerance, mobile genetic elements as prophages and the CRISPR/Cas system. The concern with antibiotic resistance is that pathogenic microorganisms may acquire antibiotic-resistant genes from the microbiota [62]. By online bioinformatics tools ResFinder and ARTS, the genome analysis showed that, no antibiotic resistance-related phenotypes or genotypes were found. LAB is known for their antibiotic sensitivity according to *in vitro* studies [63]. Most likely, the environment or stress that the bacteria were subjected to influences this finding. The resulting analysis of ARTS software related to core/essential genes confirmed all obtained from AntiSMASH. BAGEL4 tool revealed the existence of the bacteriocin-encoding genes for G2 and R1 isolate, while no hits were found for S6 isolate by these core/essential genes by ARTS, AntiSMASH and BAGEL4.

PhaBOX online tool discovered prophage sequences that were either virulent or temperate. Most identified phages belong to *Peduoviridae* and *Ackermannviridae* families that matched different suitable hosts as *L. delbrueckii* and *Streptococcus thermophilus* for both G2 and R1 strains, while no prophage sequences were found in S6 strain.

Prophages, on the other hand, contribute to the host's genome stability by modulating the individual's growth and survival in the GIT environment while also protecting the host cells from invading virulent bacteriophages and antimicrobial substances [64].

As shown in Tables 13, 14, and 15, our isolates contain CRISPR clusters in their genomes, along with Cas-genes and spacers. The existence of CRISPR clusters within a genome restricts the spread of antimicrobial resistance genes by impeding several mechanisms of horizontal transfer of genes [65]. The existence of efficient CRISPR slices provides a strain with a sequence-specific fighting barrier against phages, plasmids and insertion sequences. The CRISPR loci, with related Cas genes, also give the host strain the ability to combat itself from any invading extra-chromosomal genetic molecules [66]. This is a marker of our analyzed genome stability and a very minimal likelihood of the strain acquiring antimicrobial resistance genes, as resistant genes are generally transferred by mobile genetic elements.

## Conclusion

In conclusion, five potential probiotic isolates out of nineteen investigated isolates were examined for probiotic characteristics. The promising samples, obtained from local dairy products (yogurt and sour milk) in Egypt, were confirmed as *L. delbrueckii* subsp. *bulgaricus, Streptococcus thermophilus* and *Pediococcus acidilactici.* They demonstrated resilience to stressful environments, including NaCl, acid, bile salts, and the antimicrobial activity. The study suggests that probiotic strains resistant to antibiotics may have potential for co-administration during antibiotic treatments. Using an in-silico approach, the isolates were characterized for their physiology, safety, and efficacy. Its positive attributes are highlighted by the lack of virulence factors and pathogenicity in this study, which makes it a good choice for usage as a probiotic supplement. While the isolates lack genes conferring antibiotic resistance, the presence of mobile genetic elements and prophages may contribute to genome flexibility. R1 isolate showed the most promising probiotic properties and beneficial genetic features, followed by G2 isolate, while S6 isolate lacked some necessary genetic elements, resulting in a lower overall assessment. Further *in vivo* studies on animal and human clinical trials are needed to confirm the safety, efficacy, and health benefits of these promising isolates for probiotic use.

## Data Availability

The complete DNA sequence of the promising isolates was submitted to NCBI GenBank (submission number SUB14471673). The raw Sequencing Read Archives (SRA) was deposited in the NCBI under biosample accession numbers (G2: SAMN41534507, R1: SAMN41534508 and S6: SAMN41534509). This whole-genome sequence and all metadata for this bioproject was deposited at NCBI GenBank under (accession number PRJNA1116554).

## Abbreviations

LAB: Lactic acid bacteria
GRAS: Generally recognized as safe
FDA: The United States Food and Drug Administration
WGS: Whole genome sequence
MRS: De man, rogosa and sharpe
CLSI: Chemical Laboratory Standard Institute
OD: Optical density
MHA: Mueller-Hinton agar
MTT: 3-(4,5-dimethylthiazol-2-yl)-2, 5-diphenyltetrazolium bromide
DMEM: Dulbecco’s Modified Eagle’s ‎Medium
PBS: Phosphate buffer saline
PFU: Plaque-forming unit
PATRIC: PathoSystems Resource Integration Center
RASTtk: Rapid Annotation using Subsystem Technology tool kit
NCBI: National Center for Biotechnology Information
PGFams: PATRIC global protein families
MUSCLE: Multiple sequence alignment
RaxML: Randomized Axelerated Maximum Likelihood
CRISPR: Clustered regularly interspaced short palindromic repeats
Cas: CRISPR associated-genes
CDS: protein coding sequences
tRNA: transfer Ribo-Nucleic Acid
rRNA: ribosomal Ribo-Nucleic Acid
EC: Enzyme Commission numbers
GIt: Gastro Intestinal Tract
ZOI: zone of inhibition

## References

[1] Joint F. WHO working group report on drafting guidelines for the evaluation of probiotics in food. London: WHO and FAO; 2002. p. 16-22.

[2] Markowiak P, Slizewska K. Effects of Probiotics, Prebiotics, and Synbiotics on Human Health. Nutrients. 2017;9(9):1021. 28914794 10.3390/nu9091021 PMC5622781

[3] Mojka K. Probiotyki, prebiotyki i synbiotyki–charakterystyka i funkcje. Probl Hig Epidemiol. 2014;95(3):541-9.

[4] Cerdó T, et al. Role of microbiota function during early life on child's neurodevelopment. Trends in Food Science & Technology. 2016;57:273-88.

[5] Rossi F. "Special Issue “Functional Characterization of Lactic Acid Bacteria”." Microorganisms. 2023;11(5):1190. 10.3390/microorganisms11051190 PMC10220900 37317164

[6] Fukao M, et al. Genomic analysis by deep sequencing of the probiotic Lactobacillus brevis KB290 harboring nine plasmids reveals genomic stability. PLoS ONE. 2013;8(3):e60521. 23544154 10.1371/journal.pone.0060521 PMC3609814

[7] Germond JE, et al. Evolution of the bacterial species Lactobacillus delbrueckii: a partial genomic study with reflections on prokaryotic species concept. Mol Biol Evol. 2003;20(1):93–104. 12519911 10.1093/molbev/msg012

[8] Bolotin A, et al. Complete sequence and comparative genome analysis of the dairy bacterium Streptococcus thermophilus. Nat Biotechnol. 2004;22(12):1554–8. 15543133 10.1038/nbt1034 PMC7416660

[9] Klaenhammer TR. Genetics of bacteriocins produced by lactic acid bacteria. FEMS Microbiol Rev. 1993;12(1–3):39–85. 8398217 10.1111/j.1574-6976.1993.tb00012.x

[10] Shihata A, N.J.I.D.J., Shah. Influence of addition of proteolytic strains of Lactobacillus delbrueckii subsp. bulgaricus to commercial ABT starter cultures on texture of yoghurt. exopolysaccharide Prod survival bacteria. 2002;12(9):765–72.

[11] Grahek-Ogden D, et al. Risk assessment of Lactobacillus delbrueckii subsp. bulgaricus used as other substances. Opinion of the Panel on Biological Hazards of the Norwegian Scientific Committee for Food Safety. 2016;44:13.

[12] Serrano-Nino J, et al. Isolation and identification of lactic acid bacteria from human milk with potential probiotic role. J Food Nutr Res. 2016;4(3):170–7.

[13] Jafari B, Rezaie A, Alizadeh S. Isolation and identification of potentially probiotic bacteria from traditional dairy products of Ardabil region in Iran. Ann Biol Res. 2011;2:311–7.

[14] Vos P, et al. Bergey's manual of systematic bacteriology: Volume 3: The Firmicutes. Volume 3. Springer Science & Business Media; 2011.

[15] Pincus DH. Microbial identification using the bioMérieux Vitek® 2 system*.* Encyclopedia of Rapid Microbiological Methods. Bethesda, MD: Parenteral Drug Association, 2006. 2006: 1–32.

[16] Kumar A, Kumar D. Characterization of Lactobacillus isolated from dairy samples for probiotic properties. Anaerobe. 2015;33:117–23. 25771244 10.1016/j.anaerobe.2015.03.004

[17] Uymaz Tezel B. Preliminary in vitro evaluation of the probiotic potential of the bacteriocinogenic strain Enterococcus lactis PMD74 isolated from ezine cheese. J Food Qual. 2019;2019(1):4693513.

[18] Shaikh M, Shah G. Determination of probiotic properties of lactic acid bacteria from curd. Global J Biology Agric Health Sci. 2013;2:119–22.

[19] Vinderola CG, Reinheimer JA. Lactic acid starter and probiotic bacteria: a comparative in vitro study of probiotic characteristics and biological barrier resistance. Food Res Int. 2003;36(9–10):895–904.

[20] Bauer A, et al. Antibiotic susceptibility testing by a standardized single disk method. Am J Clin Pathol. 1966;45(4ts):493–6. 5325707

[21] Barry AL. An overview of the Clinical and Laboratory Standards Institute (CLSI) and its impact on antimicrobial susceptibility tests. Antimicrob susceptibility Test protocols, 2007: pp. 1–6.

[22] Wayne P. National committee for clinical laboratory standards (NCCLS*).* Performance standards for antimicrobial disk susceptibility testing. Twelfth informational supplement (M100-S12), 2002.

[23] Mosmann T. Rapid colorimetric assay for cellular growth and survival: application to proliferation and cytotoxicity assays. J Immunol Methods. 1983;65(1–2):55–63. 6606682 10.1016/0022-1759(83)90303-4

[24] Sirichokchatchawan W, et al. Protective effects of cell-free supernatant and live lactic acid bacteria isolated from Thai pigs against a pandemic strain of porcine epidemic diarrhea virus. Probiotics Antimicrob proteins. 2018;10:383–90. 28434154 10.1007/s12602-017-9281-y PMC7091344

[25] Abd E, Hafez MS, et al. Characterization, in-silico, and in-vitro study of a new steroid derivative from Ophiocoma dentata as a potential treatment for COVID-19. Sci Rep. 2022;12(1):5846. 35393477 10.1038/s41598-022-09809-2 PMC8991244

[26] Darwish RS, et al. Chemical profiling and unraveling of anti-COVID-19 biomarkers of red sage (Lantana camara L.) cultivars using UPLC-MS/MS coupled to chemometric analysis, in vitro study and molecular docking. J Ethnopharmacol. 2022;291:115038. 35151836 10.1016/j.jep.2022.115038 PMC8830149

[27] Civitelli L, et al. In vitro inhibition of herpes simplex virus type 1 replication by Mentha suaveolens essential oil and its main component piperitenone oxide. Phytomedicine. 2014;21(6):857–65. 24629600 10.1016/j.phymed.2014.01.013

[28] Indrayanto G, Putra GS, Suhud F. Validation of in-vitro bioassay methods: Application in herbal drug research. Profiles Drug Subst Excip Relat Methodol. 2021;46:273–307. 33461699 10.1016/bs.podrm.2020.07.005

[29] Clabaut M, et al. Draft Genome Sequence of Lactobacillus crispatus Strain V4, Isolated from a Vaginal Swab from a Young Healthy Nonmenopausal Woman. Microbiol Resour Announc. 2019;8(38). p. 10.1128/mra. 00856 – 19. 10.1128/MRA.00856-19 PMC6753271 31537667

[30] Wattam AR, et al. Improvements to PATRIC, the all-bacterial Bioinformatics Database and Analysis Resource Center. Nucleic Acids Res. 2017;45(D1):D535–42. 27899627 10.1093/nar/gkw1017 PMC5210524

[31] Brettin T, et al. RASTtk: a modular and extensible implementation of the RAST algorithm for building custom annotation pipelines and annotating batches of genomes. Sci Rep. 2015;5(1):8365. 25666585 10.1038/srep08365 PMC4322359

[32] Ondov BD, et al. Mash: fast genome and metagenome distance estimation using MinHash. Genome Biol. 2016;17(1):132. 27323842 10.1186/s13059-016-0997-x PMC4915045

[33] Davis JJ, et al. PATtyFams: Protein Families for the Microbial Genomes in the PATRIC Database. Front Microbiol. 2016;7:118. 26903996 10.3389/fmicb.2016.00118 PMC4744870

[34] Edgar RC. MUSCLE: multiple sequence alignment with high accuracy and high throughput. Nucleic Acids Res. 2004;32(5):1792–7. 15034147 10.1093/nar/gkh340 PMC390337

[35] Stamatakis A. RAxML version 8: a tool for phylogenetic analysis and post-analysis of large phylogenies. Bioinformatics. 2014;30(9):1312–3. 24451623 10.1093/bioinformatics/btu033 PMC3998144

[36] Stamatakis A, Hoover P, Rougemont J. A rapid bootstrap algorithm for the RAxML Web servers. Syst Biol. 2008;57(5):758–71. 18853362 10.1080/10635150802429642

[37] Blin K, et al. antiSMASH 7.0: new and improved predictions for detection, regulation, chemical structures and visualisation. Nucleic Acids Res. 2023;51(W1):W46–50. 37140036 10.1093/nar/gkad344 PMC10320115

[38] Mungan MD, et al. ARTS 2.0: feature updates and expansion of the Antibiotic Resistant Target Seeker for comparative genome mining. Nucleic Acids Res. 2020;48(W1):W546–52. 32427317 10.1093/nar/gkaa374 PMC7319560

[39] Bortolaia V, et al. ResFinder 4.0 for predictions of phenotypes from genotypes. J Antimicrob Chemother. 2020;75(12):3491–500. 32780112 10.1093/jac/dkaa345 PMC7662176

[40] van Heel AJ et al. BAGEL3: Automated identification of genes encoding bacteriocins and (non-)bactericidal posttranslationally modified peptides. Nucleic Acids Res, 2013. 41(Web Server issue): pp. W448–53. 10.1093/nar/gkt391 PMC3692055 23677608

[41] Shang J, et al. PhaBOX: a web server for identifying and characterizing phage contigs in metagenomic data. Bioinform Adv. 2023;3(1):vbad101. 37641717 10.1093/bioadv/vbad101 PMC10460485

[42] Couvin D, et al. CRISPRCasFinder, an update of CRISRFinder, includes a portable version, enhanced performance and integrates search for Cas proteins. Nucleic Acids Res. 2018;46(W1):W246–51. 29790974 10.1093/nar/gky425 PMC6030898

[43] Amin S, et al. Evaluation of Effect of Non Steroidal Anti-Inflammatory Drugs on Growth of Probiotics. Int J Pure Appl Sci Technol. 2014;20(1):25.

[44] Idouı T. Probiotic Potential of Lactic Acid Bacteria Isolated from Human Gut. TOJSAT. 2012;2(3):47–51.

[45] Gilliland S. Beneficial interrelationships between certain microorganisms and humans: candidate microorganisms for use as dietary adjuncts. J Food Prot. 1979;42(2):164–7. 30812336 10.4315/0362-028X-42.2.164

[46] Garriga M, et al. Selection of lactobacilli for chicken probiotic adjuncts. J Appl Microbiol. 1998;84(1):125–32. 15244067 10.1046/j.1365-2672.1997.00329.x

[47] Du Toit M, et al. Characterisation and selection of probiotic lactobacilli for a preliminary minipig feeding trial and their effect on serum cholesterol levels, faeces pH and faeces moisture content. Int J Food Microbiol. 1998;40(1–2):93–104. 9600615 10.1016/s0168-1605(98)00024-5

[48] Tulumoglu S, et al. Probiotic properties of lactobacilli species isolated from children's feces. Anaerobe. 2013;24:36–42. 24055630 10.1016/j.anaerobe.2013.09.006

[49] Botes M, van Reenen CA, Dicks LM. Evaluation of Enterococcus mundtii ST4SA and Lactobacillus plantarum 423 as probiotics by using a gastro-intestinal model with infant milk formulations as substrate. Int J Food Microbiol. 2008;128(2):362–70. 18963159 10.1016/j.ijfoodmicro.2008.09.016

[50] Gueimonde M, et al. Antibiotic resistance in probiotic bacteria. Front Microbiol. 2013;4:202. 23882264 10.3389/fmicb.2013.00202 PMC3714544

[51] Ammor MS, et al. Two different tetracycline resistance mechanisms, plasmid-carried tet(L) and chromosomally located transposon-associated tet(M), coexist in Lactobacillus sakei Rits 9. Appl Environ Microbiol. 2008;74(5):1394–401. 18192429 10.1128/AEM.01463-07 PMC2258611

[52] Noori F, TAJABADI EM, Jafari P. Growth optimization of Lactobacillus plantarum T5jq301796. 1, an Iranian indigenous probiotic in lab scale fermenter. 2016.

[53] Pan X, et al. The acid, bile tolerance and antimicrobial property of Lactobacillus acidophilus NIT. Food Control. 2009;20(6):598–602.

[54] Felifel NT, et al. Antimicrobial photodynamic therapy against Escherichia coli and Staphylococcus aureus using nanoemulsion-encapsulated zinc phthalocyanine. Microorganisms. 2023;11(5):1143. 37317117 10.3390/microorganisms11051143 PMC10222491

[55] Yüksekdağ Z, Beyatli Y, Aslim B. Determination of some characteristics coccoid forms of lactic acid bacteria isolated from Turkish kefirs with natural probiotic. LWT-Food Sci Technol. 2004;37(6):663–7.

[56] Elgohary MA-S, et al. Efficacy of Sofosbuvir plus Ledipasvir in Egyptian patients with COVID-19 compared to standard treatment: a randomized controlled trial. J Med Life. 2022;15(3):350. 35449996 10.25122/jml-2021-0175 PMC9015168

[57] Zekri A-RN et al. *Genome sequencing of SARS-CoV-2 in a cohort of Egyptian patients revealed mutation hotspots that are related to clinical outcomes.* Biochimica et Biophysica Acta (BBA)-Molecular Basis of Disease, 2021. 1867(8): p. 166154. 10.1016/j.bbadis.2021.166154 PMC8079944 33932525

[58] Bonetta L. Whole-genome sequencing breaks the cost barrier. Cell. 2010;141(6):917–9. 20550926 10.1016/j.cell.2010.05.034

[59] Patro J, et al. Development and utility of the FDA ‘GutProbe’DNA microarray for identification, genotyping and metagenomic analysis of commercially available probiotics. J Appl Microbiol. 2015;118(6):1478–88. 25766767 10.1111/jam.12795

[60] Papadimitriou K, et al. Discovering probiotic microorganisms: in vitro, in vivo, genetic and omics approaches. Front Microbiol. 2015;6:58. 25741323 10.3389/fmicb.2015.00058 PMC4330916

[61] Qureshi N, Gu Q, Li P. Whole genome sequence analysis and in vitro probiotic characteristics of a Lactobacillus strain Lactobacillus paracasei ZFM54. J Appl Microbiol. 2020;129(2):422–33. 32119175 10.1111/jam.14627

[62] Hussein WE, et al. Assessment of Safety and Probiotic Traits of Enterococcus durans OSY-EGY, Isolated From Egyptian Artisanal Cheese, Using Comparative Genomics and Phenotypic Analyses. Front Microbiol. 2020;11:608314. 33362752 10.3389/fmicb.2020.608314 PMC7759505

[63] Ramalho JB, et al. In Vitro Probiotic and Antioxidant Potential of Lactococcus lactis subsp. cremoris LL95 and Its Effect in Mice Behaviour. Nutrients. 2019;11(4):901. 31013601 10.3390/nu11040901 PMC6521076

[64] Aucouturier A, et al. Characterization of a Prophage-Free Derivative Strain of Lactococcus lactis ssp. lactis IL1403 Reveals the Importance of Prophages for Phenotypic Plasticity of the Host. Front Microbiol. 2018;9:2032. 30233519 10.3389/fmicb.2018.02032 PMC6127208

[65] Marraffini L. Sortase and the art of anchoring proteins to the envelopes of gram-positive bacteria. Microbiol Mol Biol Rev. 2006;70:192–221. 16524923 10.1128/MMBR.70.1.192-221.2006 PMC1393253

[66] Mohanan S, et al. Potential role of peptidylarginine deiminase enzymes and protein citrullination in cancer pathogenesis. Biochem Res Int. 2012;2012:p895343. 10.1155/2012/895343 PMC3457611 23019525

